# Secondary Antifungal Prophylaxis in Hematological Malignancy Patients with Previous Invasive Fungal Disease: A Retrospective Analysis

**DOI:** 10.1371/journal.pone.0115461

**Published:** 2014-12-22

**Authors:** Mingjuan Liu, Yan Li, Yongqing Zhang, Xiaoli Zhao, Bing Zhai, Qingyi Zhang, Lijun Wang, Yu Zhao, Honghua Li, Quanshun Wang, Chunji Gao, Wenrong Huang, Li Yu

**Affiliations:** 1 Department of Hematology and BMT center, Chinese PLA General Hospital, 28 Fuxing Road, Beijing 100853, China; 2 Department of Hematology, the 309th Hospital of Chinese People's Liberation Army, 17 Heishanhu Road, Beijing 100091, China; 3 Department of Hematology, Hainan Branch of Chinese PLA General Hospital, Linwang Street of Sanya City, Hainan province, 572013, China; Beth Israel Deaconess Medical Center, Harvard Medical School, United States of America

## Abstract

**Background:**

Invasive fungal disease (IFD) causes morbidity and mortality in patients with hematological malignancy. Recurrence of IFD after chemotherapy or hematopoietic stem cell transplantation (HSCT) is associated with poor prognosis. The present study aimed to investigate the efficacy of different strategies of secondary antifungal prophylaxis (SAP) for IFD and choose an appropriate SAP regimen.

**Methods:**

Clinical data of patients with previous IFD who underwent chemotherapy or HSCT between Jan 2008 and Jun 2013 were retrospectively reviewed and followed up to 180 days post-chemotherapy or HSCT. The clinical characteristics and diagnosis were analyzed according to the diagnostic criteria for IFD. The efficacy of different strategies for SAP and risk factors influencing the failure of SAP were evaluated.

**Results:**

Of the 164 patients enrolled, 121 patients received SAP regimen (73.78%), and IFD recurred in 40 patients: 16.5% (20/121) in SAP group and 46.5% (20/43) in non-SAP group. In SAP group, 58 received SAP agents which were proven effective for their previous IFD, while other 63 patients received other broad-spectrum antifungal agents. There was no significant difference in the recurrence rates between these two subgroups (13.8% (8/58) vs 19.0% (12/63), P = 0.437). The IFD recurrence rates were statistically significant between patients with allogeneic HSCT and chemotherapy or autologous HSCT (25% vs 8.2%, P = 0.013). Multivariate analysis indicated that allogeneic HSCT was the independent risk factor of IFD recurrence after SAP.

**Conclusions:**

Secondary antifungal prophylaxis is necessary to prevent IFD recurrence in patients with hematological malignancy, especially for patients in the setting of allogeneic HSCT.

## Introduction

Invasive fungal disease (IFD) is associated with significant morbidity and mortality in patients with hematological malignancy [1], [2]. The incidence of IFD has been increasing with the widespread use of high-dose chemotherapy and hematopoietic stem cell transplantation (HSCT) in recent years [3], [4]. Advancements in early diagnosis of IFD and introduction of new and more effective agents have improved the efficacy of primary antifungal treatment. It allows an increasing number of patients with hematological malignancy to undergo further chemotherapy and/or HSCT. However, IFD recurrence during an additional immunosuppressive treatment phase is very common and associated with poor outcomes. The IFD recurrence rate after intensive chemotherapy is 16% [5] and 30–50% after HSCT [6] even with appropriate treatment. The mortality among patients with recurrent IFD is as high as 88% [7].

Secondary antifungal prophylaxis (SAP) is a rational strategy for patients with previous IFD. The last published European guidelines for antifungal treatment in patients with leukemia and recipients of allogeneic HSCT pointed out that SAP should be administered to these patients with previous IFD to prevent recurrence of previous IFD or onset of a new IFD during a new at-risk phase, mainly referring to a prolonged neutropenic period induced by chemotherapy or a phase of severe immunosuppression after allogeneic HSCT [8].

Several studies reported success rates on SAP, which was proved to be effective in preventing IFD recurrence [7], [9]–[13]. However, there are no optimal preventive strategy and no specific recommendation on the selection of SAP agents, considering that the choice should be based on the causative pathogen of previous IFD and previous response to antifungal treatment [8].

Deciding an appropriate SAP regimen is challenging as the rate of recurrent IFD varies among different patients, due to the varied nature and treatment of their underlying hematological diseases, different characteristics of primary IFD, and different choice of SAP agents. While choosing the drug for SAP, patients' financial capacity, organ function, side effects of drugs, and drug-drug interactions should be taken into consideration. Consequently, it is rational that SAP should be tailored to individual patients.

In this research, the medical records of patients with hematological malignancy and history of IFD were retrospectively reviewed, and the recurrence rates of IFD with different SAP drugs were evaluated to identify the optimal strategy for SAP.

## Materials and Methods

### Patients group

One hundred and sixty-four patients with hematological malignancy having previous diagnosis of IFD (proven and probable) and receiving further chemotherapy and/or HSCT in the Department of Hematology and BMT, Chinese PLA General Hospital between Jan 2008 and Jun 2013 were reviewed retrospectively and followed up to 180 days post-chemotherapy or HSCT. Further information of patient characteristics is provided in S1 Table.

IFD was defined according to the European Organization for Research and Treatment of Cancer/Mycoses Study Group guidelines (EORTC/MSG) [14], [15].

The stage of underlying diseases was categorized. Low-risk stage was defined as first complete remission of acute lymphoblastic leukemia (ALL), acute myeloid leukemia (AML), non-Hodgkin's lymphoma (NHL) or multiple myeloma (MM), hypoplastic myelodysplastic syndrome (MDS) (refractory anemia or refractory anemia with ringed sideroblasts), first chronic phase of chronic myeloid leukemia (CML). High-risk stage was defined as those that were not included into the low-risk stage.

### Ethics statement

This study protocol was approved by the Ethics Committee of Chinese PLA General Hospital. Written informed consent was obtained from all the patients who were enrolled in this study.

### Management of underlying diseases

All patients with hematological malignancy received standard chemotherapy. Patients undergoing autologous HSCT or allogeneic HSCT received traditional conditioning regimens. Conditioning regimens included: a modified busulfan (BU)/cyclophosphamide (CY) regimen, which consisted of BU (3.2 mg·kg^−1^·d^−1^, on days −10 to −8), semustine (Me- CCNU, 250 mg/m^2^, on day −7), cytarabine (2–4 g·m^−2^·d^−1^, on days −6 and −5), and CY (60 mg·kg^−1^·d^−1^, on days −4 and −3). A modified total body irradiation/CY regimen, which consisted of total body irradiation (4–5 Gy/d) on days −8 and −7, cytarabine (3 g·m^−2^·d^−1^, on day −6), teniposide (250 mg·m^−2^·d^−1^, on day −5), and CY (60 mg·kg^−1^·d^−1^) on days −4 and −3. A modified fludarabine (Flu)/BU regimen, which consisted of Flu (30 mg·m^−2^·d^−1^, on days −10 to −6), cytarabine (1.5 mg·m^−2^·d^−1^, on days −10 and −6), and BU (3.2 mg·kg^−1^·d^−1^, on days −5 to −3). Prophylaxis for graft-versus-host-disease (GVHD) included cyclosporine, mycophenolate mofetil, and short-course methotrexate. In patients who received allo-HSCT from unrelated donors or haploidentical HSCT, antithymocyte globulin (ATG) was also required for prophylaxis for GVHD.

### SAP regimen

One hundred and twenty-one patients were administered one of the broad-spectrum antifungal agents for SAP: voriconazole (n = 45), itraconazole (n = 22), amphotericin B liposome (L-AmB) (n = 10), and caspofungin (n = 44). All SAP agents were given in accordance with the recommended doses and schedule. Patients received voriconazole with a loading dose of 6 mg/kg, every 12 h (for two doses) intravenously, followed by maintenance doses of 4 mg/kg every 12 h; or orally with a loading dose of 400 mg every 12 h (for two doses) followed by maintenance doses of 200 mg every 12 h. Patients received itraconazole with a loading dose of 200 mg, every 12 h (for four doses) intravenously, followed by maintenance doses of 200 mg every day intravenously; or orally with maintenance doses of 200 mg every 12 h. Caspofungin with a loading dose of 70 mg on the first day, followed by maintenance doses of 50 mg daily was given intravenously. L-AmB was given as a dose of 0.5–1.0 mg/kg daily intravenously, and the dosage was adjusted depending on patients' tolerability.

SAP started on the first day of conditioning or about two days before chemotherapy. SAP covered throughout the whole duration of neutropenia and terminated upon discontinuation of immunosuppression in allo-HSCT recipients or neutrophil recovery after chemotherapy or failure of SAP.

### Safety monitoring and assessment of SAP efficacy

All the patients were followed up until at least 180 days after chemotherapy or HSCT. IFD was regularly monitored through clinical symptoms, microbiological tests, and computed tomography scans.

Success of SAP was defined as the absence of documented IFD recurrence or a new IFD. Failure of SAP was defined as recurrence of previous IFD or occurrence of a new IFD. In this study, the difference between IFD recurrence and occurrence of a new IFD could not be made due to lack of sufficient data.

### Statistical analysis

Demographics and characteristics were summarized using descriptive statistics. Categorical data were compared between groups by chi-square tests or Fisher's exact test. Univariate (chi-square tests) and multivariate (logistic regressions) analyses were used to evaluate the risk factors for IFD recurrence. Multivariate analyses were performed only on variables with p≤0.25 in univariate analyses. Analyses were performed with SPSS software version 19.0 (IBM, USA). Two-tailed P values <0.05 were considered statistically significant.

## Results

### Demographics and clinical characteristics

Of the 998 patients with hematological malignancy who received treatment during the study period, 190 patients (19%) had experienced an episode of IFD. One hundred and sixty four of them were included in the study because they received further chemotherapy or HSCT after IFD was controlled. The other 26 patients were not included in the study, because 21 patients of them died of IFD and 5 gave up further treatment of underlying diseases due to partial remission of IFD. Of these 164 patients, 121 patients received SAP regimen.

The demographics and clinical characteristics of 164 enrolled patients are shown in S1 Table. The median age was 38 years old (range: 6–81 years old). Eighty four patients (51.2%) were male and 80 (48.8%) were female. The underlying diseases were acute leukemia (n = 148), NHL (n = 6), CML (n = 2), MM (n = 5), and MDS (n = 3). The underlying disease was at a low-risk stage in 102 patients, and a high-risk stage in 62 patients. Of the 164 patients, 73 received intensive chemotherapy and 91 underwent HSCT (77 underwent allogeneic HSCT, and 14 underwent autologous HSCT). Among the 77 patients who underwent allogeneic HSCT, acute GVHD occurred in 25 patients, chronic GVHD occurred in 29 patients, and cytomegalovirus (CMV) DNAemia [16] occurred in 42 patients.

All the 164 patients suffered a previous pulmonary IFD. In 2 patients, IFD was also found in liver. Proven and probable IFD were diagnosed in 16 and 148 patients, respectively. Ten of 16 proven cases were diagnosed with Aspergillus infection and 6 were having Candida infection. Ten patients with invasive pulmonary Aspergillus (IPA) infection were diagnosed by biopsy specimen of the lung, while 6 patients with invasive Candida (IC) infection by biopsy specimen of the lung or blood culture. All IPA were caused by *A. fumigatus*. Among the 6 patients with IC, 3 were caused by *C. albicans*, while 2 by *C. glabrata* and 1 by *C. tropicalis*. One hundred and thirteen of 148 patients were diagnosed with probable IPA infection and 35 with IC infection. Diagnostic criteria and results for the 164 patients are shown in Table 1. Drugs that were proved effective in previous antifungal treatments included voriconazole (n = 65), itraconazole (n = 48), L-AmB (n = 36), and caspofungin (n = 15).

**Table 1:** Diagnostic criteria and results for 164 patients with previous IFD.

Diagnostic criteria	IFD category				Total (%) n = 164
	Proven (%)		Probable (%)		
	IPA	IC	IPA	IC	
	n = 10	n = 6	n = 113	n = 35	
**Host factors**					
Neutropenia (>10 days)	10 (100)	6 (100)	45 (39.8)	17 (48.6)	78 (47.6)
T>38°C with prolonged neutropenia	10 (100)	6 (100)	45 (39.8)	12 (34.3)	73 (44.5)
Immunosuppressant	5 (50)	4 (66.7)	57 (50.4)	25 (71.4)	91 (55.5)
Previous IFD	10 (100)	6 (100)	113 (100)	35 (100)	164 (100)
With AIDS	0 (0)	0 (0)	0 (0)	0 (0)	0 (0)
GVHD	3 (30)	2 (33.3)	22 (19.5)	7 (20)	34 (20.7)
Corticosteroids#	6 (60)	2 (33.3)	46 (40.7)	15 (42.9)	69 (42.1)
**Clinical criteria**					
Halo sign	8 (80)	6 (100)	80 (70.8)	29 (82.9)	123 (75)
Air-crescent sign	5 (50)	1 (16.7)	15 (13.3)	7 (20)	28 (17.1)
Cavity	3 (30)	0 (0)	11 (9.7)	5 (14.3)	19 (11.6)
Symptoms of LRI	7 (70)	6 (100)	60 (53.1)	23 (65.7)	96 (58.5)
Permanent fever	5 (50)	4 (66.7)	64 (56.6)	25 (71.4)	98 (59.8)
**Mycological criteria**					
Positive sputum microscopy	0 (0)	/	42 (37.2)	/	42 (25.6)
Positive sputum culture	2 (20)	/	36 (31.9)	/	38 (23.2)
G test positive	3 (30)	1 (16.7)	22 (19.5)	11 (31.4)	37 (22.6)
qPCR [31] *	4 (100)	/	16 (88.9)	/	20 (90.9)
Positive blood culture	0 (0)	6 (100)	0 (0)	0 (0)	6 (3.7)
No bacterial positive	0 (0)	0 (0)	32 (28.3)	16 (45.7)	48 (29.3)
**Histology**					
Biopsy specimen of the lung	10 (100)	3 (50)	0 (0)	0 (0)	13 (7.9)

IFD, invasive fungal diseases; IPA, invasive pulmonary Aspergillosis; IC, invasive Candida; AIDS, acquired immune deficiency syndrome; GVHD, graft versus host disease; LRI, lower respiratory infections; qPCR, real-time qualitative polymerase chain reaction.

# Corticosteroid was defined as 1 mg/kg or 2 mg/kg for more than 3 weeks for the treatment of acute lymphoblastic leukemia or for the management of GVHD before IFD.

*4 proven IPA patients and 18 probable IPA patients were included in a qPCR diagnostic for IPA study, while 4 (4/4, 100%) and 16 (16/18, 88.9%) were qPCR positive respectively.

### Efficacy of SAP on prophylaxis of IFD recurrence

All patients were followed up for 180 days after chemotherapy or HSCT. The characteristics of patients in SAP group and non-SAP group are shown in Table 2. IFD recurrence occurred in 40 patients. Diagnostic criteria and results for the 40 patients are shown in an additional table [see S2 Table]. In patients receiving SAP, the recurrence rate was 16.5% (20/121); while in patients not receiving SAP, the recurrence rate was 46.5% (20/43). The recurrence rate between these two groups was statistically different (P = 0.000).

**Table 2:** Characteristics of patients in SAP group and non-SAP group.

Characteristics	SAP (n, %)	N- SAP (n, %)	*P*-value
**Gender**			0.716
Male	63 (52.1)	21 (48.8)	
Female	58 (47.9)	22 (51.2)	
**Age (years old)**			0.251
<40	65 (53.7)	31 (72.1)	
≥40	56 (46.3)	12 (27.9)	
**Underlying disease**			1.000
Acute leukemia	109 (90.1)	39 (90.7)	
Others	12 (9.9)	4 (9.3)	
**Disease stage**			0.081
Low-risk stage	80 (66.1)	22 (51.2)	
High-risk stage	41 (33.9)	21 (48.8)	
**Disease treatment**			0.257
Chemotherapy/auto-HSCT	61 (50.4)	26 (60.5)	
Allo-HSCT	60 (49.6)	17 (39.5)	
**Use of corticosteroid** *			0.974
Yes	51 (42.1)	18 (41.9)	
No	70 (57.9)	25 (58.1)	
**Duration of neutropenia**			0.838
<14 d	71 (58.7)	26 (60.5)	
≥14 d	50 (41.3)	17 (39.5)	
**Conditioning regimens**			0.880
With TBI	20 (33.3)	6 (35.3)	
Without TBI	40 (66.7)	11 (64.7)	
**Conditioning regimens**			0.926
With ATG	31 (51.7)	9 (52.9)	
Without ATG	29 (48.3)	8 (47.1)	
**Conditioning regimens**			0.674
MAC	52 (86.7)	16 (94.1)	
RIC	8 (13.3)	1 (5.9)	
**Acute GVHD**			0.761
Presence	20 (33.3)	5 (29.4)	
Absence	40 (66.7)	12 (70.6)	
**Chronic GVHD**			0.819
Presence	23 (38.3)	6 (35.3)	
Absence	37 (61.7)	11 (64.7)	
**CMV DNAemia** [16]			0.880
Presence	33 (55.0)	9 (52.9)	
Absence	27 (45.0)	8 (47.1)	
**Diagnosis of previous IFD**			0.072
Proven	15 (12.4)	1 (2.3)	
Probable	106 (87.6)	42 (97.7)	

SAP, secondary antifungal prophylaxis; auto-HSCT, autologous hematopoietic stem cell transplantation; allo-HSCT, allogeneic hematopoietic stem cell transplantation; TBI, total body irradiation; ATG, antithymocyte globulin; MAC, myeloablative conditioning; RIC, reduced-intensity conditioning; GVHD, graft-versus-host-disease; CMV, cytomegalovirus; IFD, invasive fungal disease.

*corticosteroid was defined as 1 mg/kg or 2 mg/kg for more than 3 weeks for the treatment of acute lymphoblastic leukemia or for the management of GVHD before IFD.

### Efficacy of different SAP regimens

SAP agents that the study patients received included the following: voriconazole (n = 45), itraconazole (n = 22), L-AmB (n = 10), and caspofungin (n = 44). Recurrence rates of IFD varied according to different SAP agents: 20.0% (9/45) for voriconazole, 27.3% (6/22) for itraconazole, 10.0% (1/10) for L-AmB, and 9.1% (4/44) for caspofungin. Recurrence rates of different SAP agents are shown in Table 3. There was no statistical difference in the recurrence rates of different SAP agents in all patients receiving SAP whether receiving allo-HSCT or not. Besides, no severe adverse drug reactions were reported in any subjects receiving SAP drugs. Although there was some injury of hepatic and renal function, it did not affect the SAP treatment due to the use of ancillary drugs.

**Table 3:** Recurrence rates of different SAP agents in patients receiving allo-HSCT and chemotherapy/auto-HSCT.

	Voriconazole	Itraconazole	L-AmB	Caspofungin
	(n = 45)	(n = 22)	(n = 10)	(n = 44)
Allo-HSCT	31.3% (5/16)	42.9% (6/14)	0 (0/1)	13.8% (4/29)
Chemotherapy/auto-HSCT	13.8% (4/29)	0 (0/8)	11.1% (1/9)	0 (0/15)

SAP, secondary antifungal prophylaxis; L-AmB, amphotericin B liposome; allo-HSCT, allogeneic hematopoietic stem cell transplantation; auto-HSCT, autologous hematopoietic stem cell transplantation.

### Efficacy of different SAP strategies

Among the 121 patients receiving SAP, 58 patients received previous effective antifungal drugs in primary treatment for SAP, and the remaining 63 patients were prescribed other broad-spectrum antifungal agents. There was no significant difference in the recurrence rates between these two subgroups [13.8% (8/58) vs 19.0 (12/63), P = 0.437] (Table 4). Further stratified analysis indicated that either for patients receiving allo-HSCT or receiving chemotherapy/auto-HSCT, there was also no significant difference in the recurrence rates between these two groups.

**Table 4:** Treatment of previous IFD, SAP regimens, and IFD recurrence.

SAP regimens	Previous antifungal drugs	No. of patients	No. of patients with recurrent IFD (%)
Voriconazole	Voriconazole	26	5 (19.2)
	others	19	4 (21.1)
Itraconazole	Itraconazole	13	2 (15.4)
	others	9	4 (44.4)
L-AmB	L-AmB	5	1 (20.0)
	others	5	0 (0)
Caspofungin	Caspofungin	14	0 (0)
	others	30	4 (13.3)

SAP, secondary antifungal prophylaxis; IFD, invasive fungal disease; L-AmB, amphotericin B liposome.

### Characteristics of patients with IFD recurrence

After a follow-up period of 180 days, IFD recurred in 40 patients: 20 in SAP group (16.5%) and 20 (46.5%) in non-SAP group. Among these 40 patients, 24 were male and 16 were female. The underlying diseases were AML (n = 22), ALL (n = 16), and CML (n = 2). Fifteen patients were receiving chemotherapy or autologous HSCT and 25 patients were undergoing allogeneic HSCT. The median interval time between IFD diagnosis and IFD recurrence was three months (range, 1–11 months). All the 40 patients were diagnosed as probable IFD, 20 probable IPA in SAP group and 16 probable IPA and 4 probable IC in non-SAP group. Detailed data about recurrent IFD are shown in S2 Table. Among the 20 patients with recurrent IFD in SAP group, 7 patients received single-drug therapy for recurrent IFD, 12 patients received combination therapy of two or three antifungal drugs, and 1 patient gave up therapy. By the last follow-up, 10 of the 20 patients with recurrent IFD in SAP group had died and the IFD-related mortality among patients with recurrent IFD was 45% (9/20).

### Risk factors for IFD recurrence after SAP

The following parameters were analyzed to check for their probable association with IFD recurrence: gender, age, type of underlying diseases, stage of underlying diseases, treatment of underlying diseases, duration of neutropenia, conditioning regimen, SAP agents, SAP strategy, acute GVHD, chronic GVHD, use of corticosteroid, and CMV DNAemia. Univariate analyses revealed that the recurrence rate in allo-HSCT recipients was 25%, which was much higher than that in patients receiving chemotherapy or auto-HSCT (P = 0.013), and the recurrence rate in patients with corticosteroid was much higher than patients without corticosteroid (25.5% vs 10.0%, P = 0.023).

Multivariate analyses included the following variables with a univariate P≤0.25: stage of underlying diseases, treatment of underlying diseases, use of corticosteroid, duration of neutropenia, SAP agents, and CMV DNAemia. The results showed that allo-HSCT was the independent risk factor related to recurrence rate (P = 0.046, OR: 5.094, 95%CI: 1.029–25.221).

### Patient outcome

At the end of follow-up, 38 patients had died: 29 patients were in the group of receiving SAP and 9 patients were in the non-SAP group. Patients with SAP had less recurrent IFD-related mortality than that without SAP (31.0% (9/29) vs 55.6% (5/9)). However, IFD-related mortality between these two groups was not statistically different (P = 0.245).

## Discussion

The current study retrospectively investigated the efficacy of different strategies of SAP for IFD and attempted to choose an appropriate SAP regimen. The results of this study showed that SAP can effectively prevent IFD recurrence in patients with hematological malignancy, and it is especially important for patients submitted to allogeneic HSCT. The selection of SAP agents does not have to be restricted to previous effective antifungal drugs, and other broad-spectrum antifungal drugs can also be chosen.

In recent years, many studies have assessed the efficacy of SAP and have found that SAP is efficient in preventing IFD recurrence in patients with hematological malignancy [11], [12], [17], [18]. A retrospective analysis of 48 patients from 16 bone marrow transplantation centers concluded that patients receiving SAP had less relapses of invasive Aspergillosis (IA) than did those not receiving SAP (29% vs. 59%) [7]. A meta-analysis on 239 patients with hematological malignancy indicated that IFD recurrence rates were 16% in patients with SAP and 62% in patients without SAP [19]. The present study found the similar results that the IFD recurrence rate in patients with SAP was significantly decreased compared with that in patients without SAP (16.5% vs 46.5%, P<0.001). It was also found that patients with SAP had lower recurrent IFD-related mortality than that without SAP (31.0% vs 55.6%), although there was no statistical difference (P = 0.245). The study findings showed that SAP can prevent IFD recurrence and reduce recurrent IFD-related mortality, which reflects the necessity and efficacy of SAP [7], [19].

The incidence of IFD recurrence under SAP is known to vary among different patient groups and different regions and centers. Masamoto et al. [20] reported that no IFD recurred in all of the 15 leukemia patients with SAP of voriconazole during a total of 35 courses of successive chemotherapy. Liu et al [21] reported a recurrent rate of 25.5% in their retrospective analysis of 90 allo-HSCT recipients. In the current study, an overall recurrent rate of 16.5% was observed, with 8.2% in patients receiving chemotherapy or auto-HSCT and 25% in allo-HSCT recipients. The varied recurrent rates are probably due to the varied nature and treatment of their underlying hematological malignancy, different characteristics of their previous IFD, and diverse regimens of SAP.

Many researchers have evaluated the efficacy of antifungal drugs for SAP [7], [20], [22], [23]. However, no researchers have made specific recommendations on drug selection. Fluconazole has been the standard drug for prophylaxis before and after HSCT. However, its inactivity against molds and an increasing resistance to Candida have indicated that it is unsuitable for SAP [24]. This may be the reason for no patient receiving fluconazole for SAP in the present study. Instead, a variety of broad-spectrum antifungal drugs such as voriconazole, itraconazole, AmB, and caspofungin have been found to be effective for SAP [20]–[23], [25], [26], [27]. In this study, voriconazole, itraconazole, L-AmB, and caspofungin were used for SAP. The IFD recurrence rate among different SAP drugs was compared, and it showed that there was no statistical difference in the recurrence rates of different SAP agents in all patients receiving SAP (P = 0.230), which was consistent with Cornely's result [5].

In the present study, a recurrent rate of 20% in voricinazole group was observed, which was quite higher than the previous reports [20], [23]. Many complications (such as gut GVHD occurring in allo-HSCT recipients or gastrointestinal mucositis caused by chemotherapy) might influence the bioavailability of oral voricinazole, and most of our patients received oral voricinazole for SAP. However, the recurrence rate of IFD in caspofungin group was the lowest in the study patients. Because there was no statistical difference when compared with other agents and this was a retrospective study with very few events per SAP agent group, studies with a larger sample size are required to investigate the efficacy of different SAP regimens.

So far, no relationship between antifungal drugs for previous IFD and drugs for SAP has been reported to the best of our knowledge. To know if the SAP strategies were affected by the antifungal drugs used for previous IFD, a statistical analysis was performed on the differences in recurrence rates relative to different strategies for SAP. The results showed that there was no statistical significant difference in IFD recurrence rates in choosing between their previous effective antifungal drugs and other broad-spectrum antifungal drugs for SAP. It indicates that the selection for the SAP regimens does not have to be restricted to the previous effective antifungal drugs, and other broad-spectrum antifungal drugs can also be chosen when some other limiting factors have to be considered, such as patients' economic capacity, organ function, allo-HSCT, side effects of drugs, and drug-drug interactions with the patients' concomitant medications [21].

Previous studies mainly focused on SAP in HSCT recipients [6], [10], [11], [17], [18], [21], [28], [29], whereas a fewer studies dealt with patients with chemotherapy [5], [12]. The present study included patients undergoing chemotherapy or HSCT, a comparison was made among different treatment of underlying disease. The results showed that the IFD recurrence rate in allo-HSCT recipients was much higher than that in patients receiving chemotherapy or auto-HSCT (25% vs 8.2%, P = 0.013). The probable cause is that allo-HSCT recipients have prolonged neutropenia and receive high-dose corticosteroid and long-term immunosuppression to prevent GVHD [30]. Allo-HSCT was found to be the independent risk factor for failure of SAP. Consequently, for allo-HSCT recipients, SAP appears to be more important.

The current study has several limitations. The main limitation was its retrospective nature. Confounding factors cannot be controlled effectively in retrospective studies, frequently due to biased selection of patients or treatment protocols. Another limitation of this study was that all the patients with recurrent IFD were diagnosed as probable IFD due to lack of species identification, which might affect the evaluation of SAP efficacy. This is because a relapse of IFD may be an entirely new IFD and these two types can be difficult to differentiate.

## Conclusions

In conclusion, the results of the present retrospective study indicate that SAP is effective in preventing IFD recurrence in patients with hematological malignancy, especially for patients in the setting of allogeneic HSCT. The selection for the SAP regimens does not have to be restricted to previous effective antifungal drugs, and there is considerable flexibility in drug selection for clinicians.

## Supporting Information

S1 Table **Clinical characteristics of 164 patients with previous IFD.**
(DOC)

S2 Table **Diagnostic criteria and results for the 40 patients with recurrent IFD.**
(DOC)
