# Supplementary material for: Secondary Antifungal Prophylaxis in Hematological Malignancy Patients with Previous Invasive Fungal Disease: A Retrospective Analysis
Source: PLoS One. 2014 Dec 22;9(12):e115461. doi: 10.1371/journal.pone.0115461 (PMC4274009; doi:10.1371/journal.pone.0115461)
Supplement: S1 Table — Clinical characteristics of 164 patients with previous IFD. (DOC) [file pone.0115461.s001.doc]

Table S1: Clinical characteristics of 164 patients with previous IFD

| **Demographics/characteristics** | **No. of patients (n, %)** |
| --- | --- |
| **Gender** |  |
| Male | 84 (51.2) |
| Female | 80 (48.8) |
| **Age (years old)** |  |
| < 40 | 96 (58.5) |
| ≥ 40 | 68 (41.5) |
| **Underlying disease** |  |
| Acute leukemia | 148 (90.2) |
| Others | 16 (9.8) |
| **Disease stage** |  |
| Low-risk stage | 102 (62.2) |
| High-risk stage | 62 (37.8) |
| **Disease treatment** |  |
| Chemotherapy/auto-HSCT | 87 (53.0) |
| Allo-HSCT | 77 (47.0) |
| **Acute GVHD** |  |
| Presence | 25 (32.5) |
| Absence | 52 (67.5) |
| **Chronic GVHD** |  |
| Presence | 29 (37.7) |
| Absence | 48 (62.3) |
| **CMV DNAemia^[16]^** |  |
| Presence | 42 (54.5) |
| Absence | 35 (45.5) |
| **Diagnosis of previous IFD** |  |
| Proven | 16 (9.8) |
| Probable | 148 (90.2) |
| **Primary antifungal drugs** |  |
| Voriconazole | 65 (39.6) |
| Itraconazole | 48 (29.3) |
| L-AmB | 36 (22.0) |
| Caspofungin | 15 (9.1) |

IFD, invasive fungal disease; auto-HSCT, autologous hematopoietic stem cell transplantation; allo-HSCT, allogeneic hematopoietic stem cell transplantation; GVHD, graft-versus-host-disease; CMV, cytomegalovirus; L-AmB, amphotericin B liposome.
