# Supplementary material for: Secondary Antifungal Prophylaxis in Hematological Malignancy Patients with Previous Invasive Fungal Disease: A Retrospective Analysis
Source: PLoS One. 2014 Dec 22;9(12):e115461. doi: 10.1371/journal.pone.0115461 (PMC4274009; doi:10.1371/journal.pone.0115461)
Supplement: S2 Table — Diagnostic criteria and results for the 40 patients with recurrent IFD. (DOC) [file pone.0115461.s002.doc]

Table S2: Diagnostic criteria and results for the 40 patients with recurrent IFD

| **Diagnostic criteria** | **Recurrent IFD** | | | **Total (%)** n=40 |
| --- | --- | --- | --- | --- |
|  | SAP* (n=121) | N-SAP (n=43) | |  |
|  | IPA (%) | IPA (%) | IC (%) |  |
|  | n=20 | n=16 | n=4 |  |
| **Host factors** |  |  |  |  |
| Neutropenia (>10 days) | 20 (100) | 11 (68.8 ) | 3 (75) | 34 (85) |
| T > 38°C with prolonged neutropenia | 20 (100) | 9 (56.3) | 2 (50) | 31 (77.5) |
| Immunosuppressant | 18 (90) | 10 (62.5) | 1 (25) | 29 (72.5) |
| Previous IFD | 20 (100) | 16 (100) | 4 (100) | 40 (100) |
| With AIDS | 0 (0) | 0 (0) | 0 (0) | 0 (0) |
| GVHD | 10 (50) | 3 (18.8) | 1 (25) | 14 (35) |
| Corticosteroids^#^ | 16 (80) | 5 (31.3) | 1 (25) | 22 (55) |
| **Clinical criteria** |  |  |  |  |
| Halo sign | 17 (85) | 12 (75) | 2 (50) | 31 (77.5) |
| Air-crescent sign | 12 (60) | 9 (56.3) | 1 (25) | 22 (55) |
| Cavity | 7 (35) | 6 (37.5) | 0 (0) | 13 (32.5) |
| Symptoms of LRI | 15 (75) | 11 (68.8) | 4 (100) | 30 (75) |
| Permanent fever | 16 (80) | 12 (75) | 3 (75) | 31 (77.5) |
| **Mycological criteria** |  |  |  |  |
| Positive sputum microscopy | 0 (0) | 6 ( 37.5) | / | 6 (15) |
| Positive sputum culture | 6 (30) | 4 (25) | / | 10 (25) |
| G test positive | 7 (35) | 5 (31.3) | 4 (100) | 16 (40) |
| Positive blood culture | 0 (0) | 0 (0) | 0 (0) | 0 (0) |
| No bacterial positive | 0 (0) | 4 (25) | 2 (50) | 6 (15) |
| **Histology** |  |  |  |  |
| Biopsy specimen of the lung | 0 (0) | 0 (0) | 0 (0) | 0 (0) |

IFD, invasive fungal diseases; SAP, secondary antifungal prophylaxis; IPA, invasive pulmonary Aspergillosis; IC, invasive Candida; AIDS, acquired immune deficiency syndrome; GVHD, graft versus host disease; LRI, lower respiratory infections.

*, all of the recurrent IFDs in the SAP group were all IPA.

^#^, Corticosteroid was defined as 1 mg/kg or 2 mg/kg for more than 3 weeks for the treatment of acute lymphoblastic leukemia or for the management of GVHD before IFD.
